# A study on the effect of acute hyperbaric oxygen intervention on aerobic endurance

**DOI:** 10.1186/s40101-025-00400-y

**Published:** 2025-07-17

**Authors:** Zepeng Hu, Wenjing Guo, Hao Wu

**Affiliations:** School of Sport Science and Health, Capital University of Physical Education and Sports, Beijing, 100191 China

**Keywords:** Hyperbaric oxygen, Aerobic endurance, Cardiac output, Pulmonary function, Heart rate variability, Exercise performance

## Abstract

**Introduction:**

This study aimed to explore the effects of a single mild-dose, acute hyperbaric oxygen (HBO) intervention (1.3 ATA, 100% oxygen, 60 min) on aerobic endurance, cardiac function, pulmonary function, and autonomic nervous system balance in healthy young men.

**Methods:**

Using a randomized crossover design, 14 participants received both the HBO intervention and the control condition (seated rest). For each condition, heart rate variability (HRV), cardiac function, and pulmonary function were assessed before and after the intervention, while aerobic endurance indicators—such as peak oxygen uptake (VO₂peak) and time to exhaustion (TTE)—were measured once following each condition.

**Results:**

HBO significantly decreased resting heart rate (from 63.64 ± 7.75 to 58.79 ± 7.29 bpm, Δ = −7.23%, p = 0.009), with a significant main effect of time (*F*(1,13) = 7.657, *p* = 0.016, η^2^ₚ = 0.371) and a significant time × condition interaction effect (*F*(1,13) = 4.51, *p* = 0.048, η²ₚ = 0.268). Root mean square of successive differences (RMSSD) increased from 44.50 [24.50, 59.75] to 54.00 [35.50, 67.50] (*Z* = 2.261, *p* = 0.024, *r* = − 0.604), and standard deviation of successive differences (SDSD) increased from 55.50 [31.75, 76.50] to 71.00 [55.75, 87.75] (*Z* = 2.701, *p* = 0.007, *r* = − 0.722). Both parameters also demonstrated significant differences in condition comparisons (RMSSD: *Z* = − 2.826, *p* = 0.005, *r* = − 0.755; SDSD: *Z* = − 2.796, *p* = 0.005, *r* = − 0.747). No significant changes were observed in aerobic endurance, pulmonary function, or other cardiac function parameters.

**Conclusion:**

A single mild-dose, acute HBO intervention can significantly improve resting heart rate and enhance short-term HRV parameters, suggesting a beneficial effect on parasympathetic activity. However, it does not directly enhance aerobic endurance, and long-term interventions or combined training may be needed to realize its potential benefits.

## Introduction

Aerobic endurance represents the capacity of the human body to sustain prolonged physical activity via aerobic metabolism, a fundamental determinant of athletic performance and overall health. The key determinants of aerobic endurance are the body’s capacity for oxygen delivery and utilization [1]. Therefore, enhancing cardiorespiratory function and the ability to utilize oxygen effectively are fundamental and core mechanisms for enhancing aerobic endurance. These include increases in cardiac output, enhancements in pulmonary ventilation and diffusion capacity, increased capillary density, and the optimization of skeletal muscle mitochondrial function.

Hyperbaric oxygen (HBO) intervention refers to the process of inhaling high concentrations of oxygen in an environment exceeding 1 atmosphere absolute (ATA), which necessitates complete exposure of the body and breathing 100% oxygen [2]. According to Henry’s law, HBO intervention significantly elevates the partial pressure of oxygen in the alveoli, leading to a greater amount of oxygen dissolving into the blood plasma. Theoretically, this increased oxygen availability could enhance the delivery of oxygen to exercising muscles and other tissues, potentially improving cellular function and energy production (ATP synthesis) [3]. Despite these theoretical benefits, the effects of single or acute HBO interventions on aerobic endurance and associated physiological markers remain inconclusive. Research by Cabrić and Kormanovski suggests that HBO interventions can enhance maximal oxygen uptake (VO₂max) and endurance performance [4, 5]. Other studies, such as those by Webster, Suzuki, and Hodges, have found that a single exposure to HBO does not improve aerobic endurance performance in subjects [6–8]. Notably, previous research has focused on HBO doses above 2 ATA, which may transiently cause peripheral vasoconstriction and reduced cardiac output [9], potentially affecting acute aerobic endurance performance. This may explain the contradictory results observed in previous studies. In contrast, more moderate HBO doses are sufficient to enhance oxygen supply while minimizing the risks of increased oxidative stress [10, 11]. Moreover, HBO may modulate autonomic nervous system balance by increasing heart rate variability (HRV), potentially promoting vagal activity and benefiting cardiovascular regulation and recovery [8, 12]. However, the actual effects of acute HBO interventions on aerobic endurance and their physiological mechanisms remain unclear.

In light of this, the present study adopts a randomized crossover design to compare the effects of acute HBO intervention (1.3 ATA, 100% oxygen concentration, 60 min) with a control condition (normal atmospheric environment, 60-min seated rest) on aerobic endurance in healthy young males. Simultaneously, changes in cardiac function, pulmonary function, and heart rate variability will be monitored. Our hypothesis is that acute HBO intervention may influence aerobic endurance indicators, such as VO₂max/VO₂peak and time to exhaustion (TTE), by affecting heart rate, cardiac output (CO), stroke volume (SV), pulmonary ventilation and gas exchange functions (such as forced vital capacity (FVC), forced expiratory volume in one second (FEV₁), and parasympathetic nervous activity (increasing HRV). Through this study, we aim to clarify the effects of acute HBO interventions on aerobic exercise capacity and related physiological responses.

## Materials and methods

### Participants

A total of 14 healthy male sports major students (nonsmokers) were recruited as participants in this study. The inclusion criteria were as follows: (1) The absence of the following contraindications: severe injury to the musculoskeletal system, respiratory system diseases, organic lesions of the ear, claustrophobia, history of epilepsy, chronic systemic diseases, and a history of using cardiovascular active drugs; (2) “no” responses to all questions on the Physical Activity Readiness Questionnaire (PAR-Q), indicating no exercise-related risks; and (3) the ability to complete all experimental procedures on time. After meeting the screening criteria, all participants were thoroughly informed about the purpose, procedures, and potential risks of the experiment and provided written informed consent.

### Experimental design

This study employed a 2 × 2 crossover design to evaluate the effects of acute hyperbaric oxygen (HBO) intervention on autonomic function, cardiac function, pulmonary function, and aerobic endurance. All participants underwent both intervention conditions in a randomized order, with a crossover between the two interventions to ensure a comprehensive assessment and minimize potential period or carryover effects.

#### Intervention conditions

- HBO condition (hyperbaric oxygen intervention): Participants rested inside a 1.3 ATA hyperbaric oxygen chamber for 60 min and inhaled 100% pure oxygen through an ear-hook oxygen delivery system.
- CON condition (control intervention): Participants remained seated and at rest for 60 min in a normal atmospheric environment, with no additional stimulation provided.

#### Experimental procedure

Autonomic nervous function (heart rate variability (HRV)), cardiac function, and pulmonary function were measured 10 min before and 10 min after each intervention. An aerobic endurance test was conducted immediately after each intervention.

### Crossover design and randomization

To eliminate any carryover effects and ensure the independence of measurements, a washout period of at least 1 week was maintained between the two interventions. The intervention order was assigned using a randomized block design to ensure experimental balance and control for potential period effects. Participants were randomly assigned to one of two intervention sequences: S1 (HBO → CON) or S2 (CON → HBO).

### Standardized experimental control

To ensure consistent experimental conditions, all participants were required to adhere to the following standardized control measures during the study period:

1. Abstinence from strenuous exercise and diving for 48 h prior to the experiment
2. No history of air travel within 72 h prior to the experiment to avoid the influence of barometric pressure changes on physiological indicators
3. Abstinence from consuming foods containing antioxidants (such as chocolate, red wine) for 8 h prior to the experiment to minimize the potential effects of barometric pressure changes on physiological measurements
4. Testing was scheduled at a uniform time, 2 h after a meal, on the experimental day to minimize the impact of postprandial metabolism on the experimental data.
5. Baseline measurements (including height, weight, body fat percentage) and treadmill familiarization training were completed 3 days prior to the experiment to ensure participants were familiar with the experimental procedures.

### Hyperbaric oxygen intervention procedure

**Fig. 1:**
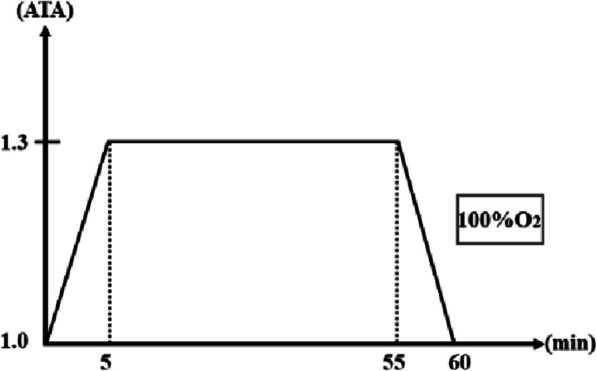
Hyperbaric oxygen intervention procedure

The hyperbaric oxygen intervention was conducted using an O_2_ ARK civil hyperbaric oxygen chamber (Shanghai Weiao Yimo Health Technology Management Co., Ltd., Shanghai, China). Participants wore an ear-hook oxygen delivery system and inhaled oxygen at a concentration of 100% or higher. The atmospheric pressure inside the chamber was maintained at 1.3 ATA, and the intervention duration was 60 min, with 5 min for both the compression and decompression phases. Throughout the entire intervention process, two researchers were always on duty outside the hyperbaric oxygen chamber to ensure the safety and monitoring of the participants (Fig. 1).

### Outcome measures

#### Heart rate variability (HRV)

This study assessed the impact of hyperbaric oxygen intervention and rest on autonomic nervous function by measuring changes in HRV before and after the intervention. Participants lay supine for 5 min in a quiet, dimly lit environment (temperature 25 ± 2 °C, relative humidity 45 ± 5%), during which they were instructed to relax, breathe spontaneously, and refrain from speaking or moving. HRV was recorded using an Omegawave autonomic nervous system assessment sensor (Omegawave Ltd., Espoo, Finland; Personal Edition; coefficient of variation 1.82%), with data captured via a chest-worn dry electrode belt. HRV assessment included frequency domain indices: Low frequency (LF), high frequency (HF), and the LF/HF ratio, as well as time domain indices: root mean square of successive differences (RMSSD), standard deviation of normal-to-normal intervals (SDNN), and standard deviation of successive differences (SDSD).

Respiration was not monitored or paced during HRV acquisition. This limitation has been acknowledged in accordance with recent methodological guidelines [13], which emphasize the influence of respiratory activity on HRV indices, particularly RMSSD and HF. Accordingly, the interpretation of parasympathetic modulation should be made with caution, as individual differences in spontaneous breathing patterns may have contributed to the observed HRV changes.

#### Pulmonary function

Pulmonary function was measured using a portable pulmonary function meter (Xeek X1, Xeek Medical Equipment Co., Ltd., Xiamen, China). The measured indices included forced vital capacity (FVC), maximal expiratory pressure (MEP), peak expiratory flow (PEF), forced expiratory volume in one second (FEV₁), forced inspiratory vital capacity (FIVC), maximal inspiratory pressure (MIP), and peak inspiratory flow (PIF). Each test was repeated three times, and the highest value was taken, ensuring a quality control grade of C or above. During the test, participants tightly sealed their lips around the mouthpiece and wore a nose clip, maintaining an upright posture with the head and neck straight, avoiding looking down or bending over. Waistbands and underwear were appropriately loosened to ensure testing accuracy. The inspiratory test required rapid and forceful inhalation to maximum inspiratory volume after complete exhalation; the expiratory test required rapid and forceful exhalation after complete inhalation, ensuring exhalation lasted ≥ 6 s and reached maximum explosive force.

#### Cardiac function

Cardiac function was assessed before and after the hyperbaric oxygen intervention or control condition using a portable noninvasive cardiac output monitor (Kaonter HT-1000, Kaonter Medical Technology Co., Ltd., Suzhou, China). This device utilizes a patented resonance frequency method. The measured indices included the following: Cardiac output (CO), cardiac index (CI), stroke volume (SV), stroke volume index (SVI), cardiac power (CP), cardiac power index (CPI), thoracic fluid content (TFC), left ventricular ejection time (LVET), pre-ejection period (PEP), ejection period (EP), total peripheral resistance (TPR), and total peripheral resistance index (TPRI). During the test, participants rested supine, remaining still and refraining from talking. Each test lasted for 5 min, and the final stable measurement value was recorded.

#### Aerobic endurance test

An incremental load protocol was performed using the Schiller PowerCube-Ergo system. The initial speed was 5.7 km/h, increasing to a maximum of 11.8 km/h every 3 min. The initial incline was 7%, gradually increasing to 14% (Table 1).

**Table 1:** Incremental load aerobic endurance test protocol

Level	Exercise stage/level	Incline (%)
1	5.7	7
2	7.1	8
3	8.0	10
4	8.4	11
5	9.4	12
6	10.5	13
7	11.8	14

The test was terminated when the participant could no longer maintain the set speed or voluntarily stopped despite strong verbal encouragement.

Criteria for judging maximal oxygen uptake or peak oxygen uptake upon test termination: All participants reached at least two of the following criteria at exhaustion. If a VO₂ plateau is observed, the value is reported as VO₂max; if no plateau is observed, the value is reported as VO₂peak. If the majority of participants do not reach a VO₂ plateau but meet two of the secondary criteria (such as *RER* ≥ 1.10, *RPE* ≥ 17, or reaching 90% of the predicted maximal heart rate), it is recommended to use VO₂peak as the evaluation index.

- VO₂ plateau (a change in VO₂ of less than 150 mL/min between consecutive stages)
- Respiratory exchange ratio (RER) ≥ 1.1
- Heart rate ≥ 90% of predicted maximum heart rate
- Rating of perceived exertion (RPE) ≥ 18 (6–20 scale) [14, 15].

#### Data acquisition

Gas metabolism data were calculated using a 30-s rolling average, and VO₂max was taken as the peak value during the last 30 s before termination. Heart rate data were continuously recorded using the Polar H10 system (Polar Electro, Kempele, Finland) at a sampling rate of 1000 Hz. The sensor was positioned at the mid-chest using an adjustable chest strap to ensure signal stability.

#### Experimental environment control

The laboratory temperature and humidity were controlled at 25 ± 2 °C and 45 ± 5%, respectively.

#### Measured indicators

Maximal oxygen uptake (VO_2_max), relative maximal oxygen uptake (VO_2_max/kg), maximal ventilation (VEmax), maximal heart rate (HRmax), maximal respiratory exchange ratio (RERmax), and time to exhaustion (TTE) were analyzed.

### Statistical methods

Continuous variables were expressed as mean ± standard deviation (*M* ± *SD*) for normally distributed data and as median [25th, 75th percentile] for non-normally distributed data. Data normality was assessed using the Shapiro–Wilk test, and homogeneity of variance was tested using the Levene test. Statistical significance was set at *p* < 0.05 (two-tailed), with post hoc comparisons adjusted using the Bonferroni correction. All analyses were performed using SPSS 26.0 (IBM, USA).

Before formal analyses, baseline equivalence between the two intervention sequences (HBO → CON and CON → HBO) was evaluated using independent-samples *t*-tests (or Mann–Whitney *U*-tests for non-normal data). Period and carryover effects, specific to crossover designs, were assessed as follows: Period effects were tested by comparing the baseline values of Period 1 and Period 2 within subjects using paired *t*-tests (or Wilcoxon signed-rank tests), to determine whether any systematic differences existed between periods that could confound the crossover comparisons. Carryover effects were assessed by calculating individual difference scores (D = Δ_HBO − Δ_CON, where *Δ* = post–pre) and comparing these D scores between intervention sequences using independent-samples *t*-tests (or Mann–Whitney *U*-tests), to identify any residual influences from the preceding intervention that might bias subsequent results. If both tests yielded *p* ≥ 0.05, data from both periods were pooled for the main analyses.

Aerobic-endurance outcomes, measured only once per intervention condition, were compared between HBO and CON using paired *t*-tests (Wilcoxon signed-rank tests for non-normal data), with effect sizes reported as Hedges’ g (0.2 = small, 0.5 = medium, 0.8 = large). For other outcomes meeting normality and homogeneity assumptions, a 2 (intervention: HBO, CON) × 2 (time: pre, post) repeated-measures ANOVA was conducted, with partial η^2^ as the effect size (< 0.06 = small, 0.06–0.14 = medium, > 0.14 = large). For non-normally distributed outcomes, change scores (*Δ* = post–pre) were analyzed using Wilcoxon signed-rank tests, comparing *Δ* against zero (within-intervention) and Δ_HBO with Δ_CON (between-intervention). Effect sizes were reported as $r=\frac{Z}{\sqrt{N}}$ (0.1 = small, 0.3 = medium, 0.5 = large). Δ (%) = (Post − Pre)/Pre × 100 was calculated using mean or median values to quantify within-group change and improve result interpretability in tables.

## Results

### Participant characteristics

Table 2 summarizes the baseline characteristics of the participants. Fourteen healthy male sports students were included (mean age: 20 ± 1.3 years; height: 176.5 ± 4.8 cm; weight: 74.8 ± 10.7 kg; skeletal muscle: 35.0 ± 4.1 kg; fat mass: 13.3 ± 5.1 kg; body fat percentage: 17.4 ± 4.5%). Baseline characteristics were balanced and met the inclusion criteria. No adverse events or dropouts occurred during the study, and all participants adhered to the standardized experimental controls.

**Table 2:** Participant information

	**Age** **(year)**	**Height** **(cm)**	**Weight** **(kg)**	**Skeletal muscle (kg)**	**Body fat** **(kg)**	**Body fat percentage (%)**
*N* = 14	20 ± 1.3	176.5 ± 4.8	74.8 ± 10.7	35.0 ± 4.1	13.3 ± 5.1	17.4 ± 4.5

Data are presented as mean ± standard deviation (*M* ± *SD*)

### Carryover effect and period effect tests

Before the formal analysis of the intervention effects, this study first evaluated potential sources of bias inherent to the crossover design, including period effects and carryover effects. In addition, baseline values of all outcome measures were compared between the two intervention sequences (HBO → CON and CON → HBO) before the first intervention. No significant differences were found, indicating good baseline equivalence between the two sequences (detailed data can be found in the “Pulmonary function evaluation,” “Cardiac function evaluation,” “Heart rate variability evaluation,” and “Aerobic endurance evaluation” sections).

Table 3 presents the results of the carryover and period effect tests. No period effects were observed for any physiological indicator after the 1-week washout period (all *P*₁ > 0.05), and no significant carryover effects were detected (all *P*₂ > 0.05). These findings indicate that the 1-week washout period was sufficient to eliminate residual intervention effects, and that the experimental period did not introduce systematic bias into the final results.

**Table 3:** Carryover effect and period effect tests

Variable	Period effect			Carryover effect		
	*p*	*t/z*	Effect size (*Hedges’ g/r*)	*p*	*t/z*	Effect size (*Hedges’ g/r*)
MEP (cm H₂O)	0.891	0.139	0.022	0.516	0.673	0.336
MIP (cm H₂O)	0.652	0.462	0.105	0.486	0.728	0.364
PEF (L · min⁻¹)	0.621	0.507	0.101	0.619	0.511	0.255
FEV₁ (mL)	0.517	-0.666	-0.091	0.700	0.401	0.201
FIVC (mL)	0.447	-0.785	-0.079	0.850	0.193	0.097
FVC (mL)	0.796	-0.264	-0.032	0.205	-1.362	-0.681
PIF (L · min⁻¹)	0.780	-0.285	-0.099	0.689	0.411	0.206
HR (bpm)	0.148	1.539	0.312	0.724	-0.361	-0.181
SV (ml)	0.614	-0.517	-0.131	0.357	-0.958	-0.479
CO (L/min)	0.342	0.987	0.246	0.618	-0.512	-0.256
SVI (mL/m²)	0.725	-0.360	-0.069	0.259	-1.185	-0.593
MAP (mmHg)	0.109	1.721	0.397	0.960	-0.051	-0.025
LVET (ms)	0.717	-0.370	-0.126	0.872	-0.164	-0.082
TFC (kΩ⁻¹)	0.995	0.006	0.002	0.775	0.292	0.146
EPCI (mmHg/min)	0.829	-0.221	-0.045	0.742	0.337	0.168
ISI (mmhg)	0.648	-0.468	-0.085	0.392	0.889	0.445
PEP (ms)*	0.242	-1.170	0.353	0.608	-0.513	-0.137
SBP (mmHg)*	0.232	-1.196	0.320	0.654	-0.448	-0.120
DBP (mmHg)*	0.209	-1.258	0.363	0.749	-0.320	-0.086
PCWP(mmHg)*	0.777	-0.283	0.076	0.749	-0.319	-0.085
RMSSD(ms)*	0.414	-0.817	0.202	0.196	-1.293	-0.346
SDSD(ms)*	0.402	-0.839	0.252	0.608	-0.513	-0.137
SDNN(ms)*	0.700	-0.385	0.107	0.847	-0.193	-0.052
LF/HF(ratio)*	0.490	-0.691	0.185	0.277	-1.086	-0.290
HF(ms²)*	0.064	-1.852	0.495	0.277	-1.086	-0.290
LF(ms²)*	0.140	-1.475	0.394	0.277	-1.086	-0.290

Variables marked with an asterisk (*) were analyzed using nonparametric tests (Wilcoxon signed-rank or Mann–Whitney *U*-tests, reporting *Z*-values), and their effect sizes were calculated as $r=\frac Z{\sqrt N}$. For normally distributed data, effect sizes were estimated using Hedges’ g. *P*₁ represents the p-value for the period effect, and *P*₂ represents the p-value for the carryover effect

### Pulmonary function evaluation

The baseline pulmonary function indicators did not differ significantly between the two intervention sequences (HBO → CON vs. CON → HBO) prior to the first intervention (all *p* ≥ 0.093).

As shown in Table 4, the 2 × 2 repeated-measures ANOVA revealed no significant time × condition interaction effects for any of the measured pulmonary function variables (maximum *F*(1,13) = 2.45, *p* = 0.14) and no significant main effects of condition (smallest *p* = 0.436, η^2^ₚ < 0.06).

**Table 4:** Effects of HBO and CON on pulmonary function

Variable	Condition	Pre	Post	Δ%	Statistics		
					Time (*p*/η^2^ₚ)	Condition (*p*/η^2^ₚ)	Interaction (*p*/η^2^ₚ)
MEP (cm H_2_O)	HBO	132.86 ± 33.42	126.14 ± 24.22	− 2.64%	0.164/0.144	0.522/0.032	0.586/0.023
	CON	133.79 ± 32.19	132.00 ± 33.80	− 0.79%			
MIP (cm H_2_O)	HBO	109.43 ± 23.01	106.86 ± 25.67	− 2.02%	0.601/0.022	0.641/0.017	0.683/0.013
	CON	105.64 ± 24.24	105.29 ± 25.67	− 0.04%			
PEF (L·min⁻^1^)	HBO	464.86 ± 95.53	424.89 ± 96.30	− 7.94%	0.084/0.212	0.701/0.012	0.141/0.159
	CON	454.14 ± 87.20	450.84 ± 74.30	+ 0.20%			
FEV1 (mL)	HBO	3988.21 ± 409.28	3921.43 ± 486.24	− 1.79%	0.064/0.239	0.571/0.025	0.885/0.002
	CON	3950.00 ± 433.46	3867.86 ± 379.32	− 3.96%			
FIVC (mL)	HBO	4285.21 ± 1 028.52	4380.86 ± 1 072.74	+ 3.30%	0.632/0.018	0.864/0.031	0.507/0.035
	CON	4324.29 ± 1039.86	4302.79 ± 1057.15	− 0.21%			
FVC (mL)	HBO	4891.00 ± 714.77	4866.00 ± 680.25	− 0.25%	0.379/0.060	0.436/0.047	0.775/0.007
	CON	4833.50 ± 600.26	4787.50 ± 602.69	− 0.91%			
PIF (L·s⁻^1^)	HBO	384.95 ± 72.90	367.22 ± 89.63	− 4.57%	0.898/0.001	0.948/0.000	0.216/0.115
	CON	364.29 ± 67.99	385.43 ± 86.48	+ 7.67%			

“Time” denotes the main effect of the measurement time point, “Condition” denotes the main effect of the intervention condition, and “Interaction” denotes the Time × Condition interaction effect

Data are expressed as mean ± standard deviation (*M* ± *SD*), *Δ* (%) is calculated as (post -pre)/pre × 100, *MEP* maximal expiratory pressure, *MIP* maximal inspiratory pressure, *PEF* peak expiratory flow, *FEV₁* forced expiratory volume in one second, *FIVC* forced inspiratory vital capacity, *FVC* forced vital capacity, *PIF* peak inspiratory flow

In terms of magnitude of change, PEF decreased by 7.9% under HBO while remaining nearly unchanged under CON (+ 0.2%); conversely, PIF decreased by 4.6% under HBO and increased by 7.7% under CON. Although these interactions were not statistically significant, both PEF and PIF demonstrated moderate effect sizes (η^2^ₚ = 0.159 and 0.115, respectively). FEV₁ decreased by ~ 3% in both conditions. Other indicators (MEP, MIP, FVC, FIVC) showed no notable changes.

Overall, a single hyperbaric oxygen exposure did not induce significant changes in pulmonary function, though the opposing moderate fluctuations in PEF and PIF warrant further investigation.

### Cardiac function evaluation

The baseline comparisons showed no significant differences between the two intervention sequences (HBO → CON vs. CON → HBO) prior to the first intervention (all *p* ≥ 0.329).

As shown in Table 5, 2 × 2 repeated-measures ANOVA revealed a significant time main effect only for HR (*F*(1,13) = 7.657, *p* = 0.016, η^2^ₚ = 0.371, large effect size) and a significant time × condition interaction effect (*F*(1, 13) = 4.759, *p* = 0.048, η^2^ₚ = 0.268, large effect size). Pairwise comparisons showed that HR decreased by 7.23% after HBO (63.64 ± 7.75 to 58.79 ± 7.29 bpm, *p* = 0.009), with no significant change in the CON condition (*p* = 0.95).

**Table 5:** Effects of HBOT and CON on cardiac function

Variable	Condition		Pre	Post	*Δ*%	Statistics		
						Time (*p*/η²ₚ)	Condition (*p*/η²ₚ)	Interaction (*p*/η²ₚ)
HR (bpm)	HBO	63.64 ± 7.75		58.79 ± 7.29*	− 7.232%	0.0162/0.371	0.287/0.087	0.048/0.268
	CON	62.71 ± 10.77		62.79 ± 8.83	0.81%			
SV (mL)	HBO	99.93 ± 8.48		102.86 ± 8.82	3.37%	0.675/0.014	0.777/0.006	0.112/0.183
	CON	102.43 ± 10.67		101.43 ± 11.95	− 0.68%			
CO (L·min^⁻1^)	HBO	6.43 ± 0.76		6.12 ± 0.54	− 4.10%	0.052/0.260	0.471/0.041	0.376/0.061
	CON	6.42 ± 0.72		6.35 ± 0.77	− 0.82%			
SVI (mL·m^⁻2^)	HBO	52.76 ± 5.96		54.34 ± 6.44	3.38%	0.626/0.019	0.714/0.011	0.144/0.157
	CON	54.09 ± 7.53		53.79 ± 8.29	− 0.22%			
MAP (mmHg)	HBO	88.00 ± 5.87		87.29 ± 5.30	− 0.43%	0.216/0.115	0.784/0.006	0.442/0.046
	CON	89.64 ± 6.78		86.79 ± 7.89	− 3.04%			
LVET (ms)	HBO	304.57 ± 35.94		322.21 ± 41.22	6.66%	0.615/0.020	0.619/0.020	0.103/0.192
	CON	321.50 ± 30.98		310.63 ± 32.50	− 3.00%			
TFC (kΩ^⁻1^)	HBO	30.86 ± 2.70		31.61 ± 5.09	3.62%	0.563/0.026	0.592/0.023	0.343/0.069
	CON	32.50 ± 4.42		30.81 ± 2.97	− 3.94%			
EPCI (mmHg·min^⁻1^)	HBO	62.99 ± 16.52		61.95 ± 14.18	2.09%	0.984/0.000	0.298/0.083	0.355/0.066
	CON	65.22 ± 16.54		66.38 ± 14.32	3.66%			
ISI (ms^2^)	HBO	102.46 ± 29.84		98.33 ± 22.44	%	0.772/0.007	0.481/0.039	0.444/0.046
	CON	103.61 ± 30.03		105.10 ± 23.79	4.78%			
Indicator	Condition	Pre		Post	Δ%	Within *P*/*r*	Between* p*/*r*	
PEP (ms)	HBO	100.00 [92.00, 109.00]		102.00 [99.00, 108.00]	2.00%	0.457/0.199	0.431/− 0.210	
	CON	104.00 [99.00, 108.00]		102.00 [88.00, 109.00]	− 1.92%	0.779/0.075		
PCWP (mmHg)	HBO	11.90 [11.30, 13.40]		11.50 [11.10, 13.65]	− 3.36%	0.637/0.126	0.432/− 0.210	
	CON	13.40 [11.25, 13.65]		11.40 [11.00, 13.25]	− 14.93%	0.172/− 0.365		
SBP (mmHg)	HBO	127.00 [120.75, 128.50]		124.00 [120.75, 128.75]	− 2.36 %	0.576/− 0.150	0.413/− 0.219	
	CON	127.50 [116.50, 130.00]		123.50 [109.75, 129.00]	− 3.89%	0.083/− 0.463		
DBP (mmHg)	HBO	68.00 [64.75, 75.25]		67.50 [63.75, 71.25]	− 0.74%	0.599/− 0.141	0.683/− 0.109	
	CON	71.50 [68.25, 80.00]		71.50 [62.00, 75.25]	0.00%	0.362/− 0.244		

Values are shown as *mean* ± *SD* for normal data and median [P25 P75] for non-normal data, *Δ* (%) is calculated as (post- pre)/pre × 100, based on the mean or median accordingly

*HR*, heart rate, *SV* stroke volume, *CO* cardiac output, *SVI* stroke-volume index, *MAP* mean arterial pressure, *PEP* pre-ejection period, *LVET* left-ventricular ejection time, *TFC* thoracic fluid content, *SBP* systolic blood pressure, *DBP* diastolic blood pressure, *EPCI* ejection-phase cardiac index, *ISI* inotropic state index, *PCWP* pulmonary capillary wedge pressure, (*p*/η^2^ₚ) indicates the *p*-value and the partial eta-squared effect size from repeated-measures ANOVA, Within *P*/*r* denotes the *p*-value (*P*) and effect size (*r*) from Wilcoxon signed-rank tests comparing pre- and post-intervention values within each condition (HBO or CON), Between *P*/*r* denotes the *p*-value (*P*) and effect size (r) comparing the change scores (*Δ*) between HBO and CON using Wilcoxon signed-rank tests

^*^Significant difference from pre‑intervention at* p* < 0.05

Cardiac output (CO) decreased by 1.7% after HBO and by 0.8% after CON, with a marginal time effect (*F*(1,13) = 4.57, *p* = 0.052, η^2^ₚ = 0.260, medium effect size) without statistical significance. PCWP increased by 7.9% after HBO and decreased by 3.1% after CON, although not significant (*Z* = 0.472, *p* = 0.432, *r* = − 0.210, small-to-medium effect size).

Other cardiac indicators (SV, SVI, MAP, LVET, TFC, EPCI, ISI) showed no significant main or interaction effects (all *p* ≥ 0.05, η^2^ₚ ≤ 0.26). Wilcoxon signed-rank tests confirmed no significant within- or between-condition differences for these indicators (all *p* ≥ 0.08).

Overall, these results suggest that a single acute HBO exposure significantly reduced resting HR, with no clear impact on other cardiac function parameters.

### Heart rate variability evaluation

The baseline comparisons of heart rate variability showed no significant differences between the two intervention sequences (HBO → CON vs. CON → HBO) prior to the first intervention (all *p* ≥ 0.329).

As shown in Table 6, RMSSD significantly increased in the HBO condition, with median values rising from 44.50 [24.50, 59.75] to 54.00 [35.50, 67.50] (*Z* = 2.261, *p* = 0.024, *r* = − 0.604; large effect). The condition comparison also showed a significant difference (*Z* = − 2.826, *p* = 0.005, *r* = − 0.755). Similarly, SDSD significantly increased from 55.50 [31.75, 76.50] to 71.00 [55.75, 87.75] in the HBO condition (*Z* = 2.701, *p* = 0.007, *r* = − 0.722, large effect size), and the condition comparison revealed a significant difference (*Z* = − 2.796, *p* = 0.005, *r* = − 0.747).

**Table 6:** Effects of HBO and CON on heart rate variability

Indicator	Condition	Pre	Post	*Δ*%	Within-condition *P*/*r*	Between-condition *p*/*r*
RMSSD	HBO	44.50 [24.50, 59.75]	54.00 [35.50, 67.50]*	21.35%	0.024/− 0.604	0.005/− 0.755
(ms)	CON	46.00 [37.25, 54.75]	42.50 [35.75, 50.75]	− 7.61%	0.375/− 0.237	
SDSD	HBO	55.50 [31.75, 76.50]	71 [55.75, 87.75]**	27.93%	0.007/− 0.722	0.005/− 0.747
(ms)	CON	59 [47.00, 70.25]	57 [48.50, 72.50]	− 3.39%	0.826/− 0.059	
SDNN	HBO	60.00 [41.50, 84.75]	65.5 [35.75, 87.25]	9.17%	0.801/− 0.067	0.683/− 0.109
(ms)	CON	53.00 [47.50, 97.25]	69.50 [47.00, 85.00]	31.13%	0.826/0.059	
LF/HF	HBO	1.215 [0.570, 1.923]	1.240 [0.660, 1.948]	2.06%	0.730/0.092	0.925/− 0.025
(ratio)	CON	1.425 [0.725, 1.930]	1.205 [0.893, 1.740]	− 15.44%	0.875/0.042	
HF	HBO	278.50 [139.25, 734.25]	542.50 [238.25, 895.00]	94.79%	0.245/0.311	0.433/0.210
(ms^2^)	CON	363.00 [214.00, 591.25]	297.50 [138.25, 586.00]	18.04%	0.470/− 0.193	
LF	HBO	419.50 [122.25, 794.50]	493.50 [250.25, 1165.75]	17.64%	0.397/0.226	0.47/− 0.193
(ms^2^)	CON	377.00 [283.50, 816.75]	361.50 [218.00, 695.50]	4.11%	0.826/− 0.059	

Values are shown as median [P25, P75], *Δ* (%) = (after-pre)/pre × 100, calculated based on the median

Within P/r denotes the *p*-value (*P*) and effect size (*r*) from Wilcoxon signed-rank tests comparing pre- and post-intervention values within each condition (HBO or CON), Between *P*/*r* denotes the *p*-value (*P*) and effect size (*r*) comparing the change scores (*Δ*) between HBO and CON using Wilcoxon signed-rank tests

*RMSSD* root mean square of successive differences, *SDSD* standard deviation of successive differences, *SDNN* standard deviation of normal-to-normal intervals, *LF/HF* low-frequency to high-frequency ratio, *HF* high-frequency power, *LF* low-frequency power

*Significant difference from pre-intervention at *p* < 0.05

**Significant difference from pre-intervention at *p* < 0.01

SDNN, LF/HF, HF, and LF did not show significant changes within conditions or between conditions (all *p* > 0.05). Notably, HF increased in the HBO condition from 278.50 [139.25, 734.25] to 542.50 [238.25, 895.00] (*Z* = 1.162, *p* = 0.245, *r* = 0.311; moderate effect), but this was not statistically significant, and the condition comparison was also non-significant (*Z* = − 0.785, *p* = 0.433, *r* = − 0.210). LF also exhibited a numerical increase in the HBO condition (*Z* = 0.847, *p* = 0.397, *r* = 0.226), which did not reach statistical significance.

In summary, these results suggest that a single acute HBO intervention can significantly improve short-term HRV time-domain parameters (RMSSD, SDSD), potentially reflecting enhanced parasympathetic modulation and improved cardiac autonomic regulation.

### Aerobic endurance evaluation

In this study, 14 participants each completed two incremental exercise tests, yielding a total of 28 test sessions. Of these, six tests during the first session met the criterion for a VO₂ plateau (ΔVO₂ < 150 mL/min) and were classified as VO₂max, while the remaining eight tests were reported as VO₂peak. In the second session, six tests similarly achieved a VO₂ plateau, with the other eight not reaching this threshold. Across both sessions, 12 out of 28 tests (42.9%) exhibited a VO₂ plateau and were reported as VO₂max, while the remaining 16 tests—although not meeting the plateau criterion—fulfilled at least two secondary maximal effort criteria (*RER* ≥ 1.10, *HR* ≥ 90% of age-predicted maximum, *RPE* ≥ 18), justifying the reporting of VO₂peak.

As shown in Table 7, no statistically significant differences were found in any aerobic endurance indicator between the HBO and CON conditions. VO₂peak/kg was nearly identical between conditions (*t*(13) = 0.144, *p* = 0.888, Hedges’ *g* = 0.036). Similarly, VO₂peak (*t*(13) = 0.414, *p* = 0.686, Hedges’ *g* = 0.104), VEpeak (*t*(13) = − 1.136, *p* = 0.276, Hedges’ *g* = − 0.286), and HRpeak (*t*(13) = 0.130, *p* = 0.899, Hedges’ *g* = 0.033) also showed no meaningful differences. TTE was comparable between conditions as well (*t*(13) = 0.456, *p* = 0.656, Hedges’ *g* = 0.155). All effect sizes were small or negligible, indicating that a single mild-dose HBO exposure did not acutely enhance aerobic endurance in this study.

**Table 7:** Effects of HBO and CON on aerobic endurance indicators

Variable	HBO	CON	*p*	Hedges’ *g*
VO₂peak (L·min^−¹^)	3.85 ± 0.43	3.83 ± 0.43	0.686	0.104
VO₂peak/kg (mL·kg^−¹^·min^−¹^)	52.14 ± 7.08	52.01 ± 6.60	0.888	0.036
VEpeak (L·min^⁻¹^)	139.46 ± 17.11	141.95 ± 16.32	0.276	−0.286
HRpeak (bpm)	192 ± 8	191 ± 10	0.899	0.033
TTE (s)	665 ± 173	657 ± 162	0.656	0.155

Data are expressed as mean ± standard deviation (*M ± SD*)

Differences between interventions were analyzed using paired-samples *t*-tests. Hedges’ *g* was reported as the effect size

*VO₂peak* peak oxygen uptake, *VO₂peak/kg* peak oxygen uptake per kilogram, *VEpeak* peak ventilation, *HRpeak* peak heart rate, *TTE* time to exhaustion

## Discussion

This study demonstrated that a single, acute HBO intervention did not produce significant improvements in aerobic endurance indicators among the participants. No significant differences were observed between the HBO and control conditions in VO₂peak, TTE, HRpeak, or VEpeak, suggesting that a single 60-min exposure at 1.3 ATA of moderate-dose hyperbaric oxygen was insufficient to immediately enhance maximal aerobic capacity in healthy, trained individuals. This finding is consistent with the results reported by McGavock et al. [6–8, 16].

Regarding cardiovascular function, the single acute HBO intervention significantly decreased resting HR, but did not induce significant changes in other key cardiovascular parameters such as CO and SV. Previous studies have shown that inhaling high-concentration oxygen can increase peripheral vascular resistance and lower HR [17], resulting in a slight decrease in CO. In healthy individuals, acute oxygen inhalation typically reduces CO by approximately 10%, while systemic vascular resistance increases, and mean arterial pressure remains largely unchanged, with no enhancement in overall oxygen delivery [9]. This aligns with the present study’s findings: although HBO significantly reduced HR, it did not result in significant increases or decreases in CO (in this study, CO showed a slight decline with a moderate effect size that did not reach statistical significance), nor were there significant changes in blood pressure. These observations collectively suggest that a single HBO session does not substantially enhance cardiac pumping capacity or systemic oxygen delivery. Since maximal aerobic capacity is largely limited by CO and skeletal muscle oxygen utilization, the acute HR reduction observed here likely reflects an oxygen-concentration-driven “adaptive balance” response, rather than a true enhancement of cardiac function.

For pulmonary function, HBO exposure did not produce statistically significant changes in lung ventilation or lung volumes in the healthy young men. Although FEV₁ approached significance for a time main effect (*p* = 0.064), overall, there were no significant changes in FVC, FEV₁, MEP, MIP, FIVC, or PIF. While PEF and PIF displayed opposite moderate effect size fluctuations (PEF: HBO decreased 7.9%; PIF: HBO decreased 4.6% and CON increased 7.7%), these did not reach statistical significance. These findings suggest that a single, acute HBO exposure does not elicit substantial short-term alterations in pulmonary function. This is consistent with previous studies showing that lung function in healthy individuals typically requires long-term training adaptations or pathological changes to see significant shifts. For instance, Hadanny et al. [18] conducted a prospective study involving 60 HBO sessions (2 ATA, 100% oxygen, 5 days/week, with 5-min air breaks every 20 min) and found slight improvements in PEF and FVC without significant adverse effects. Other studies also confirm that even higher doses of HBO (2–2.4 ATA) do not lead to oxygen toxicity [19]. Therefore, the present study further supports the notion that short-term HBO does not negatively impact pulmonary function or notably enhance lung functional reserve. The directional differences and moderate effect sizes in PEF and PIF indicate that these subtle fluctuations may result from individual variability or temporary changes in respiratory muscle control.

Regarding autonomic nervous system activity, HRV time-domain indices (e.g., RMSSD, SDSD) were significantly increased following HBO intervention, indicating enhanced parasympathetic modulation. Previous studies have shown that inhalation of high-concentration oxygen in normobaric or hyperbaric environments can increase the high-frequency components of HRV and decrease the LF/HF ratio, reflecting elevated vagal activity [20]. This oxygen-induced “vagal excitation” may arise from chemoreceptor and baroreceptor reflexes: high oxygen levels cause peripheral vasoconstriction and a sharp increase in arterial oxygen content, leading to reduced HR through baroreceptor-mediated feedback involving the vagus nerve [21]. It is worth noting that although the frequency-domain indices (LF/HF, HF, LF) in this study did not reach statistical significance, HF still demonstrated a moderate effect size increase under the HBO condition, suggesting consistency in this autonomic adjustment trend. Overall, this HRV elevation pattern is generally considered an indicator of enhanced autonomic activity during recovery or relaxation in exercise physiology and is commonly interpreted as a marker of good recovery in competitive sports [22]. However, our findings also indicate that this resting HRV improvement did not translate into immediate enhancements in aerobic endurance (e.g., VO₂peak, TTE) during exercise. In other words, acute HBO more prominently influenced the physiological state during recovery rather than the energy supply capacity during exercise. It is important to note that during HRV measurement in this study, respiratory frequency was neither controlled nor recorded, even though respiratory frequency can significantly affect HRV time-domain indices (particularly RMSSD). Therefore, the post-HBO increase in HRV observed here may partly reflect individual variations in breathing patterns. This has been acknowledged as an important limitation in our study, and future research should consider standardizing or synchronously recording respiratory frequency to better elucidate the neuroregulatory mechanisms of HRV changes and their coupling with respiratory patterns [13].

From the perspective of muscle metabolism, it was initially hypothesized that HBO might improve muscle oxygen utilization efficiency, but the results did not support this idea. The lack of differences in VO₂peak during the exhaustion test indicates that the muscles’ ability to take up and utilize oxygen at peak levels was similar across conditions. While the hyperbaric oxygen environment increases the body’s oxygen delivery and diffusion capacity, if the skeletal muscle’s oxygen utilization capacity (such as mitochondrial respiratory enzyme function) has not changed, the maximal oxygen uptake is still limited by the preexisting cardiac output and muscle metabolic limits. Studies have pointed out that when athletes do not experience exercise-induced hypoxemia under normal conditions, additional oxygen supply will not further enhance VO₂peak [23]. Oxygen supplementation can only improve VO₂peak and endurance when exercise is limited by insufficient oxygen supply (e.g., when arterial oxygen saturation decreases during exercise) [24]. Additionally, considering the adaptation of oxygen-utilizing enzymes, a single HBO session is unlikely to alter mitochondrial function, which may be one of the chronic adaptations needed to enhance aerobic capacity. Literature reports that under long-term HBO exposure, the body undergoes repeated high-oxygen/normal-oxygen alternations, known as the hyperbaric oxygen/high-oxygen-low-oxygen paradox [25, 26], which may activate molecular pathways similar to hypoxic training (such as HIF-1α, Nrf2), thereby regulating oxidative stress, energy metabolism, and adaptive responses. Hadanny et al. [27] conducted a double-blind, randomized, placebo-controlled study showing that 40 sessions of HBO (2.0 ATA, 100% oxygen for 1 h) significantly increased VO₂peak and anaerobic threshold, along with improvements in skeletal muscle mitochondrial respiration and mitochondrial content. Research by Burgos et al. [28] also indicated that a 3-week HBO intervention (2.0 ATA, 21% O₂) did not increase oxidative stress markers and improved endurance performance in young soccer players. This suggests that HBO’s effect on aerobic capacity is more evident in long-term adaptations, rather than from a single session. On the other hand, long-term HBO may also improve oxidative stress balance and increase antioxidant enzyme activity, thus protecting mitochondrial function [2], while acute HBO intervention may cause a temporary imbalance in the oxidative-antioxidative system [29]. Moreover, a single HBO intervention is more likely suited for postexercise recovery. Research has shown that postexercise HBO intervention can effectively alleviate exercise-induced inflammation and muscle damage [30], allowing athletes to perform better in subsequent high-intensity exercises after a short recovery period [31].

### Study limitations

This study has several important limitations that should be noted:

1. The sample size of 14 participants, while sufficient to detect some significant changes, may be relatively small to reveal more subtle effects or interactions. In addition, no a priori power analysis was conducted, which is an important limitation when interpreting nonsignificant findings.
2. During HRV testing, we did not control for or record actual breathing frequency. Since respiratory frequency has a significant impact on HRV time-domain indices (especially RMSSD), we cannot exclude the possibility that the observed increases in HRV following HBO were partially influenced by individual differences in breathing patterns.
3. Due to the nature of the HBO intervention, participant blinding was not possible, which may have introduced placebo effects or biases from subjective effort. Future research with larger sample sizes, more rigorous controls (e.g., sham chamber conditions to achieve double-blinding), and investigations into the long-term effects of HBO interventions will be needed to comprehensively assess its potential impact on exercise performance and related physiological systems.

## Conclusion

A single mild-dose hyperbaric oxygen (HBO) exposure (1.3 ATA, 100% oxygen, 60 min) reduced resting heart rate and significantly increased resting heart rate variability (RMSSD and SDSD), reflecting a short-term enhancement of parasympathetic activity within the autonomic nervous system. However, this acute intervention did not produce any immediate improvements in aerobic endurance, and the observed enhancements in autonomic function at rest did not translate into improved aerobic performance during exhaustive exercise. Future studies should focus on the long-term effects of HBO interventions, adaptive responses in different populations, and the potential for integrating HBO into training and recovery strategies.

## Data Availability

No datasets were generated or analysed during the current study.

## Abbreviations

HBO: Hyperbaric oxygen
VO₂max: Maximal oxygen uptake
HR: Heart rate
HRV: Heart rate variability
RMSSD: Root mean square of successive differences
SDSD: Standard deviation of successive differences
SDNN: Standard deviation of normal-to-normal intervals
LF: Low frequency
HF: High frequency
LF/HF: Low-frequency to high-frequency ratio
PEF: Peak expiratory flow
FVC: Forced vital capacity
FEV₁: Forced expiratory volume in one second
MIP: Maximal inspiratory pressure
MEP: Maximal expiratory pressure
PIF: Peak inspiratory flow
CO: Cardiac output
SV: Stroke volume
MAP: Mean arterial pressure
PEP: Pre-ejection period
LVET: Left ventricular ejection time
SVI: Stroke volume index
RER: Respiratory exchange ratio
RPE: Rating of perceived exertion
FIVC: Forced inspiratory vital capacity
TFC: Thoracic fluid content
ISI: Inter-stroke interval
PCWP: Pulmonary capillary wedge pressure
ATA: Atmospheres absolute
ANOVA: Analysis of variance
VO₂peak: Peak oxygen uptake
VEmax: Maximal ventilation
HRmax: Maximal heart rate
RERmax: Maximal respiratory exchange ratio
VO₂peak/kg: Peak oxygen uptake per kilogram of body weight
TTE: Time to exhaustion
